# Pro-Resolving Macrophage-Induced IL-35^+^ but Not TGF-β1^+^ Regulatory B Cell Activation Requires the PD-L1/PD-1 Pathway

**DOI:** 10.3390/ijms26115332

**Published:** 2025-06-01

**Authors:** Guoqin Cao, Takumi Memida, Shengyuan Huang, Elaheh Dalir Abdolahinia, Sunniva Ruiz, Sahar Hassantash, Jayant Ari, Satoru Shindo, Jiang Lin, Toshihisa Kawai, Xiaozhe Han

**Affiliations:** 1 Department of Oral Science and Translational Research, College of Dental Medicine, Nova Southeastern University, 3200 South University Drive, Fort Lauderdale, FL 33328, USA; 2 Department of Stomatology, Beijing Tongren Hospital, Capital Medical University, Beijing 100005, China

**Keywords:** macrophage, interleukin 35, TGF-beta, regulatory B cell, PD-L1

## Abstract

The interaction between immune regulatory cells, such as regulatory B cells (Breg) and pro-resolving macrophages (M2 macrophages), plays an important role in the restoration of immune homeostasis during inflammation. PD-L1 is one of the effector molecules that mediates the immune regulation function of M2 macrophages. The activation of PD-L1/PD-1 signaling promotes the differentiation of Breg. Previous studies have shown that Breg promoted M2 macrophage polarization and enhanced their function, but little is known about the regulatory function of M2 macrophages on Breg differentiation. This study aims to determine the effect of M2 macrophages on Breg induction and the potential mechanism in vitro. Bone-marrow-derived macrophages were isolated from wild-type (WT) mice and polarized into M2 using IL-4/IL-13. To investigate the role of PD-L1/PD-1 in M2 macrophage-induced Breg differentiation, spleen B cells were isolated from WT or PD-1 knockout (KO) mice and co-cultured with either naïve (M0) or M2 macrophages for 48 h with or without trans-well inserts. The expression of IL-10, IL-35, and TGF-β1 in B cells was evaluated by flow cytometry and immunofluorescence staining. Recombinant PD-L1 was used to stimulate activated B cells, followed by the detection of IL-35 and TGF-β1. The results show that there was no significant difference in IL-10 expression among all groups. However, IL-35 and TGF-β1 expression in B cells was significantly increased in the M2+B, but not in M0+B, compared to B cells alone. Notably, such increases were diminished when M2 and B cells were separated by trans-well inserts. IL-35 expression was not significantly changed when B cells from PD-1 KO mice were co-cultured with M2 compared to the control. However, TGF-β1 expression was significantly increased when PD-1 KO B cells were co-cultured with M2 compared to the control. IL-35 expression in activated B cells was increased upon stimulation with PD-L1. However, TGF-β1 expression in activated B cells was increased regardless of the PD-L1 availability. This study demonstrates that pro-resolving macrophage-induced IL-35^+^ but not TGF-β1^+^ regulatory B cell activation requires the PD-L1/PD-1 pathway.

## 1. Introduction

The host immune response functions as a double-edged sword in autoimmune diseases such as multiple sclerosis [1], rheumatoid arthritis [2], and systemic lupus erythematosus [3], as well as in infectious diseases such as periodontitis [4]. While moderate immune responses protect the host, excessive immune responses disrupt the immune microenvironment, leading to tissue damage. Therefore, promoting the restoration of immune homeostasis is crucial for mitigating disease progression and minimizing tissue damage. It has been widely reported that immunoregulatory cells, such as regulatory B cells (Breg), regulatory T cells (Treg), and pro-resolving macrophages (M2 macrophages), play an important role in regulating the immune response, promoting tissue repair, and maintaining immune homeostasis [5,6,7,8,9]. Investigating the interactions among these immune regulatory cells is crucial to elucidating the mechanisms through which they mediate their regulatory functions.

Breg is characterized by the expression of anti-inflammatory cytokines that contribute to its regulatory functions, such as IL-10, IL-35, and TGF-β1 [5,6,10]. The most studied cytokine was IL-10, which attenuates harmful immune responses and promotes tolerance [11]. However, growing evidence shows that B-cell-derived IL-35 also contributes to the maintenance of immune homeostasis [6,12]. IL-35, a member of the IL-12 family composed of two subunits, Ebi3 and IL-12a, is a newly defined anti-inflammatory cytokine that could induce the expansion of Breg and Treg [13,14] but suppress the function of Th17 and osteoclast [15,16]. It has been reported that mice with B-cell-restricted deficiency of IL-35 lose their ability to recover from experimental autoimmune encephalomyelitis (EAE) [6], and Breg mediates the immunosuppression in pancreatic tumor by reducing natural killer (NK) cells through IL-35 [17]. TGF-β1 is another immune regulatory cytokine of Breg. It has been demonstrated that Breg promotes graft survival by promoting Treg development via TGF-β production [18], and B-cell-specific TGF-β1 deficiency leads to earlier onset of EAE [10]. Published studies have also shown a positive correlation between IL-35 and TGF-β1 expression [19,20].

M2 macrophages, one of the major subtypes of macrophages in inflammation, play an important role in resolving inflammation, promoting tissue repair, and maintaining homeostasis [8,9]. Our previous studies demonstrated that Breg promoted M2 macrophage polarization and that Breg alleviated periodontal inflammation in vivo via macrophage-mediated mechanisms, while Breg enhanced the pro-resolving function of M2 macrophages through cell–cell interaction in vitro [21,22]. These findings suggested that the interaction between M2 macrophages and Breg is essential for restoring tissue homeostasis and preventing chronic inflammatory conditions. However, little is known about the potential role of M2 macrophages in Breg differentiation.

The PD-L1/PD-1 pathway is an important immunoregulatory target. PD-L1/PD-1 activation has been reported to upregulate the expression of IL-10, IL-35, and TGF-β1. Treatment with PD-L1 increased IL-10 expression in B cells [23]. It has been demonstrated that PD-L1^+^ cells are positively correlated with IL-35^+^ cells in non-small cell lung cancer [24], and the activation of the PD-L1/PD-1 pathway increased the expression of IL-35 in PBMC [25]. TGF-β1 expression in T cells was decreased after PD-1 blockade [26]. PD-1 was expressed in B cells, and PD-1^hi^ B cells suppress the immune response of T cells [27,28]. These findings suggest that PD-L1/PD-1 activation may also contribute to Breg differentiation. A recent study showed that PD-L1^+^CD206^+^ macrophages express high levels of PD-L1 and inhibit immune response through PD-L1 [29]. Other studies have indicated that PD-L1 increased the expansion of Breg [30,31]. These studies suggest a potential link between M2 macrophages and the differentiation of Breg. This study aims to explore the role of M2 macrophages in Breg differentiation and their potential molecular mechanisms.

## 2. Results

### 2.1. M2 Macrophages Promoted the Expression of IL-35 and TGF-β1 in B Cells

**Figure 1:**
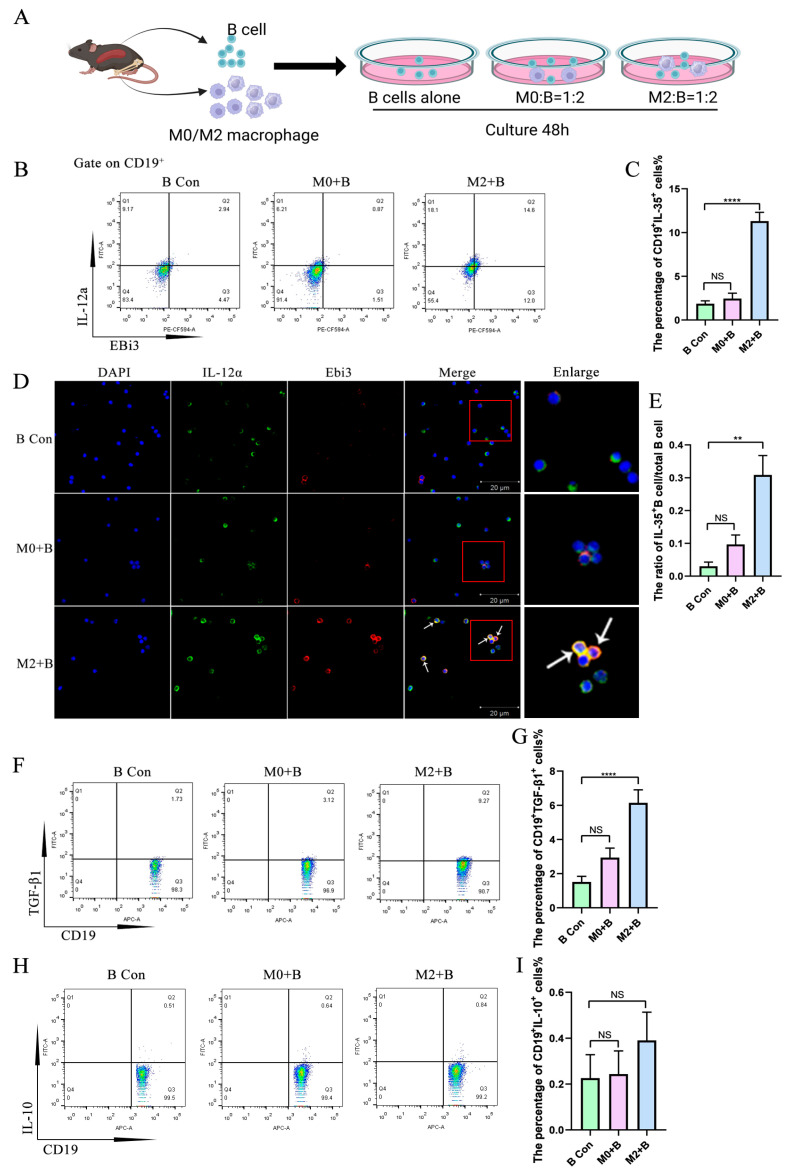
M2 macrophages promoted the expression of IL-35 and TGF-β1 in B cells. B cells were co-cultured with macrophages for 48 h, followed by flow cytometry and immunofluorescence staining detection. (**A**) A schematic of B cells co-cultured with macrophages. (**B**) IL-35 (Ebi3^+^IL-12a^+^) expression in B cells detected by flow cytometry. (**C**) Statistical data of IL-35 expression in B cells (*n* = 12). (**D**) IL-35 expression in B cells detected by immunofluorescence staining (The white arrow points to IL-35^+^Breg; The enlarged image on the right shows the portion of the red box). (**E**) Statistical data of IL-35 expression in B cells (*n* = 7). (**F**) TGF-β1 expression in B cells detected by flow cytometry. (**G**) Statistical data of TGF-β1 expression in B cells (*n* = 12). (**H**) IL-10 expression in B cells detected by flow cytometry. (**I**) Statistical data of IL-10 expression in B cells. Statistical significance was analyzed by one-way ANOVA followed by Tukey’s test and Dunnett’s *t*-test. NS: no significant difference. ** *p* < 0.01; **** *p* < 0.0001.

B cells were co-cultured with M0 or M2 for 48 h, and both immunofluorescence staining and flow cytometry results show that IL-35 expression in B cells significantly increased after co-culture with M2 macrophages, but not after co-culture with M0 macrophages, compared with the control group (Figure 1B–E). TGF-β1, another cytokine with regulatory function, was increased in B cells after co-culture with M2 macrophages but not after co-culture with the M0 macrophage group compared with the control group (Figure 1F,G). However, IL-10 expression in B cells was not increased by co-culture with M2 macrophages nor with M0 macrophages compared to the control group (Figure 1H,I).

### 2.2. M2 Macrophages Promoted IL-35 and TGF-β1 Expression in B Cells Through Direct Cell–Cell Contact

**Figure 2:**
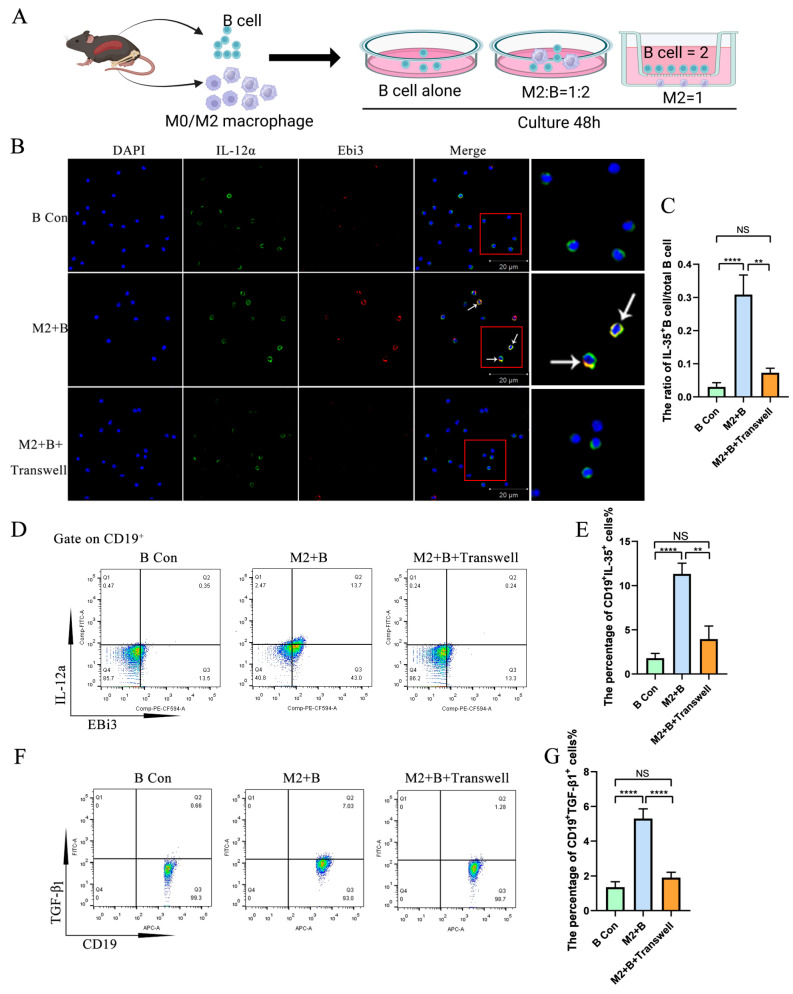
M2 macrophage-induced IL-35 and TGF-β1 expression in B cells requires direct cell–cell contact. B cells were co-cultured with M2 macrophages with or without a trans-well insert, followed by flow cytometry and immunofluorescence staining. (**A**) A schematic of B cells co-cultured with macrophages with a trans-well insert. (**B**) A typical picture of IL-35 expression in B cells detected by immunofluorescence staining (The white arrow points to IL-35^+^Breg; The enlarged image on the right shows the portion of the red box). (**C**) Statistical data of IL-35 expression in B cells detected by immunofluorescence staining (*n* = 7). (**D**) IL-35 (Ebi3^+^IL-12a^+^) expression in B cells detected by flow cytometry. (**E**) Statistical data of IL-35 expression in B cells detected by flow cytometry (*n* = 6). (**F**) TGF-β1 expression in B cells detected by flow cytometry. (**G**) Statistical data of TGF-β1 expression in B cells (*n* = 6). Statistical significance was analyzed using a one-way ANOVA followed by Tukey’s test and Dunnett’s *t*-test. NS: no significant difference. ** *p* < 0.01; **** *p* < 0.0001.

To further investigate the mechanism of IL-35 and TGF-β1 expression in B cells induced by M2 macrophages, a trans-well was used to physically separate M2 macrophages and B cells during co-culture. The results show that the expression of IL-35 and TGF-β1 in B cells dramatically increased in the B cells directly co-cultured with the M2 macrophage group compared with the control group and in the B cells co-cultured with M2 macrophages with a trans-well insert group (Figure 2). However, both IL-35 and TGF-β1 expression in B cells had no significant difference between the B cells co-cultured with M2 macrophages with a trans-well insert group and the control group (Figure 2).

### 2.3. M2-Induced Upregulation of IL-35 but Not TGF-β1 in B Cells Requires PD-1

**Figure 3:**
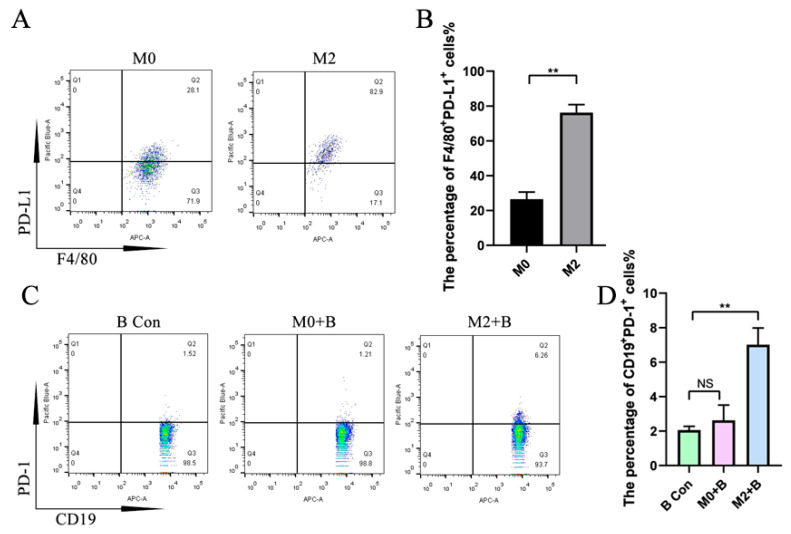
The expression of PD-L1 increased in M2 macrophages, and M2 macrophages promoted PD-1 expression in B cells. BMDM was stimulated with IL-4/IL-13 for 48 h, and B cells were co-cultured with macrophages for 48 h, followed by flow cytometry detection. (**A**) PD-L1 expression in BMDM detected using flow cytometry. (**B**) Statistical data of PD-L1 expression in BMDM detected using flow cytometry (*n* = 4). (**C**) PD-1 expression in B cells detected using flow cytometry. (**D**) Statistical data of PD-1 expression in B cells (*n* = 6). PD-L1 expression was analyzed using Student’s *t*-test, and PD-1 expression was analyzed using the Mann–Whitney U test. NS: no significant difference; ** *p* < 0.01.

PD-L1 and PD-1 play an important role in the expression of IL-35 and TGF-β1. To further study the mechanism of the effect of M2 macrophages on IL-35 expression in B cells, first, we detected PD-L1 expression in macrophages and PD-1 expression in B cells using flow cytometry. The results show that the expression of PD-L1 increased in M2 macrophages compared with M0 macrophages (Figure 3A,B). Its receptor PD-1 expression in B cells was significantly increased after co-culture with M2 macrophages but not when co-cultured with M0 macrophages compared with the control group (Figure 3C,D).

**Figure 4:**
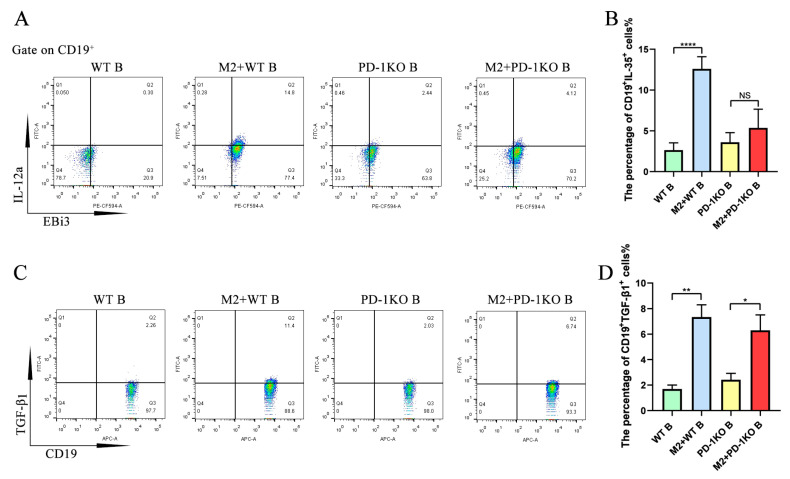
M2 macrophage-induced upregulation of IL-35 but not TGF-β1 in B cells requires PD-1. Wild-type or PD-1 KO mice-derived B cells were co-cultured with macrophages, followed by flow cytometry detection. (**A**) IL-35 expression in B cells detected using flow cytometry. (**B**) Statistical data of IL-35 expression in B cells detected using flow cytometry (*n* = 7). (**C**) TGF-β1 expression in B cell detected using flow cytometry. (**D**) Statistical data of TGF-β1 expression in B cells detected using flow cytometry (*n* = 7). Statistical significance was analyzed using one-way ANOVA followed by Tukey’s test. NS: no significant difference. * *p* < 0.05, ** *p* < 0.01, and **** *p* < 0.0001.

To investigate the function of the PD-L1/PD-1 axis on M2 macrophage-induced IL-35 and TGF-β1 expression in B cells, B cells derived from WT mice or PD-1 KO mice were cultured alone or co-cultured with M2 macrophages for 48 h. The results show that the expression of CD19^+^IL-35^+^ B cells was significantly increased in the WT B cells co-cultured with the M2 macrophage group compared with the WT B cell control group (Figure 4A,B). However, the expression of CD19^+^IL-35^+^ in B cells showed no significant difference between the PD-1 KO B cells co-cultured with the M2 macrophage group and the PD-1 KO B cell control group (Figure 4A,B). The expression of CD19^+^TGF-β1^+^ in B cells was not only significantly increased in the WT B cells co-cultured with the M2 macrophage group compared with the WT B cell control group, but it was also increased in the PD-1 KO B cells co-cultured with the M2 macrophage group compared with the PD-1 KO B cell control group (Figure 4C,D). These results show that the M2 macrophage-induced upregulation of CD19^+^IL-35^+^ B cells was PD-1-dependent, but the upregulation of CD19^+^TGF-β1^+^ B cells was independent of PD-1.

### 2.4. PD-L1 Promotes the Upregulation of IL-35 but Not TGF-β1 in Activated B Cells

**Figure 5:**
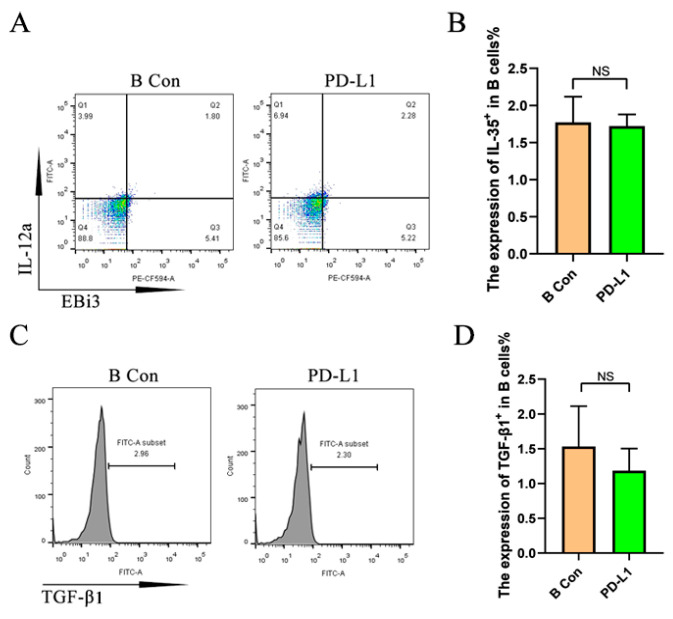
The expression of IL-35 and TGF-β1 in naive B cells was not increased by PD-L1 stimulation. Naïve B cells were stimulated with PD-L1 for 48 h, followed by flow cytometry detection. (**A**) IL-35 expression in B cells detected by flow cytometry. (**B**) Statistical data of IL-35 expression in B cells detected by flow cytometry (*n* = 6). (**C**) TGF-β1 expression in B cells detected by flow cytometry. (**D**) Statistical data of TGF-β1 expression in B cells detected by flow cytometry (*n* = 6). Statistical significance was analyzed using Student’s *t*-test. NS: no significant difference.

To further study the function of PD-L1/PD-1 in how M2 macrophages induce Breg, recombinant PD-L1 was used to stimulate the naïve B cells. The results show that the expression of IL-35 and TGF-β1 in B cells had no significant difference between the PD-L1 stimulation group and the control group (Figure 5).

**Figure 6:**
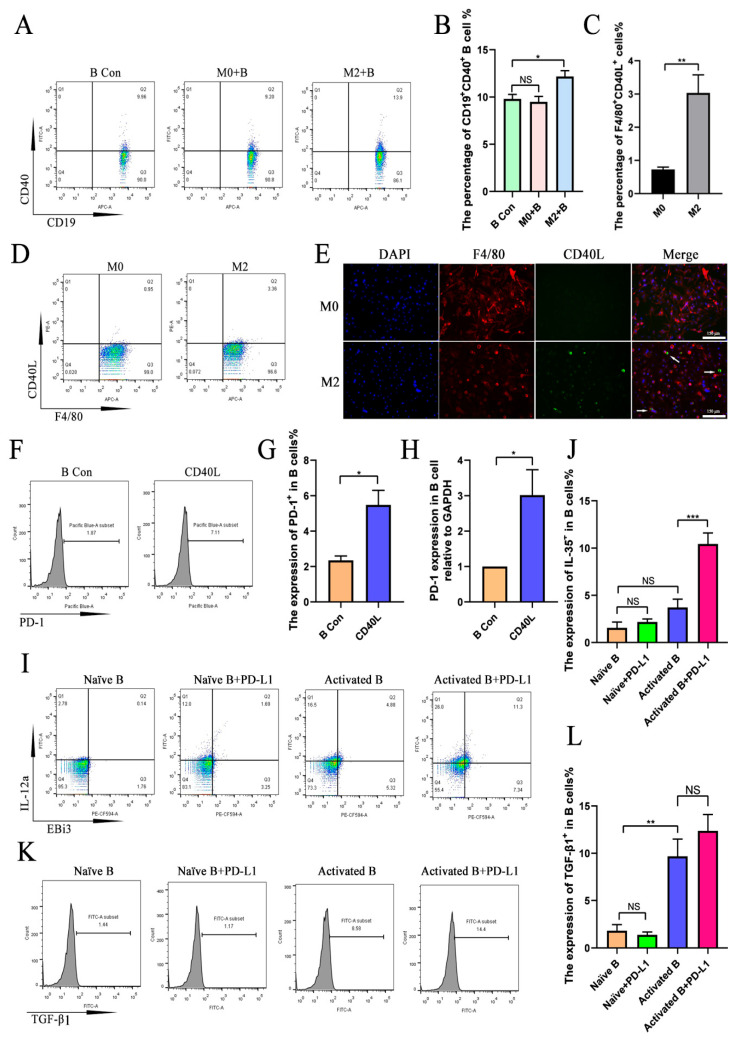
The upregulation of IL-35 but not TGF-β1 expression in activated B cells requires PD-L1/PD-1 activation. Naïve B cells were co-cultured with M0 or M2 macrophages for 48 h followed by flow cytometry detection of the expression of CD40 in B cells. (**A**) CD40 expression in B cells detected by flow cytometry. (**B**) Statistical data of CD40 expression in B cells detected by flow cytometry (*n* = 4). The macrophages were stimulated with IL-4/IL-13 for 48 h, followed by flow cytometry and immunofluorescence staining detection of the expression of CD40L in macrophages. (**C**) Statistical data of CD40L expression in macrophages detected by flow cytometry (*n* = 6). (**D**) CD40L expression in macrophages detected by flow cytometry. (**E**) A typical picture of CD40L expression in macrophages detected by immunofluorescence staining. Naïve B cells were stimulated with CD40L for 48 h, followed by flow cytometry and RT-PCR detection of the expression of PD-1 in B cells. (**F**) PD-1 expression in B cells detected by flow cytometry. (**G**) Statistical data of PD-1 expression in B cells detected by flow cytometry (*n* = 3). (**H**) *PD-1* expression in B cells detected by RT-PCR. Naïve B cells or CD40L-activated B cells were stimulated with PD-L1 for 48 h, followed by flow cytometry detection of the expression of IL-35 and TGF-β1 in B cells. (**I**) IL-35 expression in B cells detected by flow cytometry. (**J**) Statistical data of IL-35 expression in B cells (*n* = 5). (**K**) TGF-β1 expression in B cells detected by flow cytometry. (**L**) Statistical data of TGF-β1 expression in B cells (*n* = 5). Student’s *t*-test was used to analyze the difference between two independent groups, and a one-way analysis of variance followed by Tukey’s test was used to carry out multiple data comparisons. NS: no significant difference. * *p* < 0.05, ** *p* < 0.01, and *** *p* < 0.001.

The interaction of CD40L/CD40 is important for B cell activation in vitro. The results show that the expression of CD40 in B cells was increased in the B cells co-cultured with the M2 macrophage group but not in the B cells co-cultured with the M0 macrophage group compared to the control group (Figure 6A,B). Meanwhile, CD40L expression was significantly increased in M2 macrophages compared with M0 macrophages (Figure 6C–E). Thus, recombinant CD40L was used to activate B cells in this study. First, we detected the effect of CD40L on PD-1 expression in B cells. The results show that PD-1 expression in B cells significantly increased in the CD40L stimulation group compared with the control group (Figure 6F–H). Next, recombinant PD-L1 was used to stimulate CD40L-actived B cells. The results show that the expression of IL-35 in activated B cells significantly increased in the PD-L1-treated group compared to the activated B cell group (Figure 6I,J). IL-35 expression in B cells showed no significant difference between the activated B cell group and the naïve B cell group (Figure 6I,J). The expression of TGF-β1 in B cells was significantly increased in the activated B cell group compared with the naïve B cell group (Figure 6K,L). However, the expression of TGF-β1 in the PD-L1-stimulated activated B cells increased, but there was no significant difference in TGF-β1 expression in B cells between the activated B cell group and the activated B+PD-L1 group (Figure 6K,L). These results show that PD-L1 upregulated IL-35^+^ expression in activated B cells but not in naïve B cells, and the expression of TGF-β1 in activated B cells did not require PD-L1 stimulation in vitro.

## 3. Discussion

Accumulating evidence indicates that tissue damage associated with various inflammation and autoimmune diseases is partly driven by dysregulated host immune responses, such as rheumatoid arthritis, systemic lupus erythematosus, and periodontitis [2,3,4]. Studying the cross-talk of immune regulatory cells, such as pro-resolving macrophages (M2 macrophages) and Breg, is important to understand the mechanism of how to promote the restoration of immune homeostasis, which is essential for terminating disease progression. This study demonstrated for the first time that M2 macrophages promote the expression of IL-35^+^ Breg and TGF-β1^+^ Breg through direct cell–cell contact in vitro.

Breg has many cell surface markers in addition to many cytokines, such as IL-10, IL-35, and TGF-β1, which may be categorized into different subsets. For example, Koichi Yanaba’s published study revealed that the major phenotype of IL-10^+^ Breg after LPS stimulation is CD19^+^CD1d^hi^CD5^+^ [32]; another study showed that PD-1^hi^ B cells play a significant role in suppressing immune responses in the hepatocellular carcinoma environment, and the major phenotype of Breg is CD5^hi^CD^24−/+^ CD27^hi/+^CD38^dim^ [28]. Jamie G. Evans’s group found that B220^+^CD21^hi^CD23^+^ Breg suppressed the induction of arthritis [33]. Although many cell surface markers can be used as “indicators” of Breg, no specific phenotypic markers for the Breg lineage have been identified, so Breg is still termed for its immunomodulatory function. It has been reported that Breg promotes Treg expression either in an IL-10-dependent or non-IL-10-dependent manner [34,35]. This study showed that M2 macrophages could not upregulate IL-10 expression in B cells but upregulate IL-35 and TGF-β1 expression in B cells, which have also been shown to promote Treg expression [14,36,37]. Therefore, in this study, M2-stimulated B cells were co-cultured with T cells to confirm the regulatory function of M2 macrophage-stimulated B cells. The results show that M2 macrophage-stimulated B cells promoted the expression of CD4^+^TGF-β1^+^ and CD4^+^CD25^+^FOXP3^+^ Treg cells (Supplementary Figure S2). Treg cells play an important role in controlling autoimmune disease [38]. This result demonstrates that B cells acquire an immunoregulatory function after M2 induction, suggesting that M2 macrophages can induce Breg production.

IL-35 is a newly defined anti-inflammatory cytokine that promotes the expansion of Treg and Breg but suppresses the function of Th17 cells and inhibits osteoclast expression [13,14,15,16]. Our study shows that the expression of IL-35 was increased in CD19^+^ B cells after co-culture with M2 macrophages, which was consistent with other studies showing that Breg is one of the sources of IL-35 [6,17]. TGF-β1 is another important regulatory cytokine of Breg [39]. This study also shows that the expression of TGF-β1 in B cells was increased by M2 macrophages. These results are consistent with other studies showing that the expression of IL-35 and TGF-β1 is positively correlated with each other [19,20]. It has been reported that the B-cell-restricted deficiency of IL-35 diminish their ability to recover from EAE and that B cells reduce the proliferation of NK cells in tumors through IL-35 [6,17]; other published research shows that B-cell-specific TGF-β1 deficiency leads to the earlier onset of EAE [10]. These studies demonstrate that IL-35 and TGF-β1 are powerful regulatory cytokines of Breg. However, the expression of IL-35 and TGF-β1 in B cells induced by M2 macrophages was diminished by the trans-well insert (Figure 2). These results suggest that the receptor–ligand interaction plays an important role in M2 macrophage-induced IL-35^+^ Breg and TGF-β1^+^ Breg expression.

The interaction between PD-L1 and its receptor PD-1 was well-known to play an important role in immune regulation [40,41]. The activation of PD-L1/PD-1 signaling promotes the differentiation of Breg [23]. Our results show that the expression of PD-L1 was increased in IL-4/IL-13-induced M2 macrophages, and there was an increase in PD-1 expression in B cells after co-culture with M2 macrophages but not with M0 macrophages. It has been reported that PD-1^hi^ B cells were identified as a novel Breg population [28]. In addition, PD-L1/PD-1 activation promotes the expression of IL-35 in PBMC [25]. Taken together with the results of these published studies, our results suggest that the M2 macrophage upregulation of IL-35 in B cells might be related to the PD-L1/PD-1 axis. To investigate whether direct cell–cell contact, which was required for IL-35 expression in B cells that was induced by M2 macrophages, was related to the interaction of PD-L1 and PD-1, we co-cultured B cells derived from wild-type C57 BL/6J mice or PD-1 KO mice with M2 macrophages. The results show that M2 macrophages lose the ability to upregulate the expression of IL-35 in PD-1KO B cells. This result suggests that PD-1 was required in M2-induced IL-35^+^ Breg. PD-L1, a ligand for PD-1, has been reported to upregulate the expression of IL-35 in PBMC [25]. Our results show that PD-L1 expression in M2 macrophages was increased. To further investigate the function of PD-L1/PD-1 interaction in M2-induced IL-35^+^ expression in B cells, recombinant PD-L1 was used as a stimulator in this study. Contrary to our expectations, IL-35 expression in B cells was not upregulated by PD-L1 stimulation alone. The reason for this may be that PD-1 was expressed at a higher level in active B cells [27] but at lower levels in naïve B cells, which explains why PD-L1 failed to stimulate IL-35 expression in naïve B cells. This was confirmed by our results, where a low level of PD-1 expression in B cells was seen before co-culture with M2 macrophages. These results suggest that factors other than PD-L1 were required for B cell activation by M2 macrophages in vitro. M2 macrophage-induced TGF-β1^+^ expression in Breg also required direct cell–cell contact. However, the expression of TGF-β1 in B cells induced by M2 macrophages was increased in both WT mice-derived B cells and PD-1KO mice-derived B cells after co-culture with M2 macrophages. Although a decrease in TGF-β1 expression in T cells upon PD-1 blockade has been reported [26], unlike T cells, TGF-β1 expression in M2 macrophage-induced Breg is independent of PD-1.

Co-stimulation is important for B cell activation and maturation in vitro. The results of this study show that the expression of CD40 in B cells was increased by M2 macrophages, which is an important co-stimulation factor for B cell activation [42,43]. This suggests that CD40 might be involved in the activation of B cells during co-culture with M2 macrophages. Our results also show an increase in CD40L expression in M2 macrophages compared to M0 macrophages, which is supported by other studies showing that CD40L was detected in macrophages [44,45]. This study shows that IL-35 expression in B cells induced by M2 macrophages occurred due to direct cell–cell contact and was PD-1 dependent. Moreover, an increase in PD-1 expression in B cells after co-culture with M2 macrophages is shown in this study. PD-1 was expressed at a higher level on active B cells [27], and the interaction between CD40 and its ligand CD40L plays an important role in promoting B cell activation in vitro [5,6,42,46]. Thus, recombinant CD40L was used to activate naïve B cells in vitro in this study. Our results show that PD-1 was increased in CD40L-activated B cells, which is supported by another study showing that PD-1 expression in B cells was upregulated by stimulation with CD40L [47]. This could be one of the mechanisms for increased PD-1 expression in B cells after co-culture with M2 macrophages, also providing an explanation for how IL-35^+^ expression in Breg could be induced by M2 macrophages in a PD-1-dependent pathway but did not increase in naïve B cells after PD-L1 stimulation.

Since PD-1 expression was increased in activated B cells, we investigated whether PD-L1 could increase IL-35 expression in activated B cells. The results show that IL-35 expression in activated B cells was significantly increased by stimulation with PD-L1. These results suggest that M2 macrophages promoted the activation of B cells and M2 upregulated IL-35 expression in activated B cells via PD-L1/PD-1 activation. BAFF, another co-stimulator for B cell differentiation, has been reported to induce IL-35^+^ expression in Breg through TACI/NF-κB axis, as well as their expression in macrophages [48,49,50]. This is another candidate molecule related to M2’s regulation role in IL-35^+^ expression in Breg, which needs further study. In addition, this study shows that the expression of TGF-β1 was increased by CD40L stimulation without PD-L1, further confirming that M2 macrophage-induced TGF-β1^+^ expression in Breg does not require the involvement of PD-1. This result is supported by another study, which showed that CD40L/CD40 promotes TGF-β1 in B cells [51]. Although the expression of TGF-β1 in B cells was increased by interaction with M2 macrophages, the mechanism still needs further study. The results in this study show that M2 macrophages promoted B cell activation, and M2 macrophages upregulated IL-35 expression in activated-B cells via the PD-L1/PD-1 pathway; additionally, the upregulation of the expression of TGF-β1 in M2-induced Breg does not require PD-L1/PD-1 activation. While mainly focused on the characterization of IL-35^+^/TGF-β1^+^ regulatory B cells, the current study has some limitations that warrant further investigations. Firstly, it remains to be determined whether there is an overlap in the expression of IL-10, IL-35, and TGF-β1 in induced Breg, where two or three of these cytokines are concurrently expressed by the same cell. This clarification will be useful to define distinct Breg subsets associated with these cytokine markers. Secondly, it is recognized that IL-35-producing plasma B cells, also known as i35-Bregs or regulatory B cells, are a subset of B cells that play a critical role in regulating immune responses [6]. It will be of great interest to determine plasma B cell (CD138) differentiation during the M2 macrophage–B cell interaction, which was not included in the current study. Thirdly, the ultimate test of the role of pro-resolving macrophages on Breg induction and the potential mechanism must come from observations of cell–cell interactions in the context of the immune microenvironment. Therefore, future in vivo studies monitoring and evaluating the dynamic changes in targeted cells are required to obtain definitive answers to these questions.

In summary, this study demonstrates that M2 macrophages induce IL-35^+^ Breg and TGF-β1^+^ Breg expression through direct cell–cell contact and in differential molecular pathways in vitro. It provides novel insights into the mechanism underlying the immunomodulatory role of macrophages. Taken together with our previous studies, this study indicates that there is a feedback loop between M2 macrophages and Breg, which contributes to a better understanding of the immune cell cross-talk in inflammation regulation. However, the regulatory function of M2 macrophages on Breg in vivo and whether M2 macrophages orchestrate inflammation resolution through Breg functions still warrant further investigation.

## 4. Materials and Methods

### 4.1. Animal

Eight-to-ten-week-old wild-type C57BL/6J (weight 20–24 g) and PD-1 knockout (PD-1 KO, weight 20–24 g) mice were purchased from Jackson Laboratory (Bar Harbor, ME, USA). All mice were housed under specific pathogen-free conditions. All the procedures were approved by the Institutional Animal Care and Use Committee (IACUC) of Nova Southeastern University (Approval number: 2022.02.XH1).

### 4.2. BMDM Isolation and Culture

Bone-marrow-derived macrophage (BMDM) was obtained from the femur and tibia of C57 BL/6J mice. Red blood cells were removed with 3 mL/mouse ACK lysing buffer (Gibco, Gland Island, NY, USA) at room temperature for 3 min, and the bone marrow cells were then cultured in complete Iscove’s Modified Dulbecco’s Medium (IMDM) (Gibco, Gland Island, NY, USA) supplemented with 10% FBS (Gibco, Gland Island, NY, USA), 1% penicillin-streptomycin (Gibco, Gland Island, NY, USA), and 5 × 10^−5^ M 2-Mercaptoethanol (Gibco, Gland Island, NY, USA). The bone marrow cells were differentiated into M0 macrophages by treatment with 40 ng/mL recombinant mouse M-CSF (Biolegend, San Diego, CA, USA) for 4 days. Then, BMDM was seeded into 24-well plates with a density of 2 × 10^5^ cells/well. To generate M2 macrophages, M0 macrophages were stimulated with 20 ng/mL IL-4 (PeproTech, Cranbury, NJ, USA) and 20 ng/mL IL-13 (PeproTech, Cranbury, NJ, USA) for 48 h. All BMDM cells were cultured in a humidified incubator with 5% CO_2_ at 37 °C.

### 4.3. B Cell Isolation

Primary spleen B cells were separated from C57 BL/6J mice or PD-1 KO mice with a Pan B cell isolation kit (Miltenyi Biotec, Charlestown, MA, USA) under the manufacturer’s instructions. Briefly, spleen cells were incubated with a biotin cocktail for 10 min at 4 °C to label non-B cells and then incubated with anti-biotin microbeads for 15 min at 4 °C. B cells were collected by passing through LD columns (Miltenyi Biotec, Charlestown, MA, USA) in a magnetic field (Miltenyi Biotec, Charlestown, MA, USA). Yield with >95% purity was used for the subsequent experiment.

### 4.4. Co-Culture

For co-culture experiment, using 4 × 10^5^ cells/well, B cells were cultured alone or co-cultured with 2 × 10^5^ cells/well of M2 macrophages with or without a trans-well insert for 48 h in a 24-well plate, and 50 ng/mL PMA (Sigma-Aldrich, Saint Louis, MO, USA), 500 ng/mL ionomycin (MCE, Monmouth Juanction, NJ, USA), and 1 μM monensin (Biolegend, San Diego, CA, USA) were added at the final 5 h. The following groups were set up: WT mice-derived B cells cultured alone (B con or WT B), WT mice-derived B cells co-cultured with M0 macrophages (M0 + B), WT mice-derived B cells co-cultured with M2 macrophages (M2 + B or M2 + WT B), WT mice-derived B cells co-cultured with M2 macrophages with a trans-well insert (M2 + B + trans-well), PD-1 KO mice-derived B cells cultured alone (PD-1KO B), and PD-1 KO mice-derived B cells co-cultured with M2 macrophages (M2 + PD-1 KO B).

### 4.5. PD-L1 Stimulation

CD40 is a co-stimulatory molecule on B cells. The interaction between CD40L and CD40 promotes B cell activation and maturation in vitro [35,36]. Naïve B cells were activated with 1 μg/mL CD40L in this study. Then, the naïve B cells and CD40L-activated B cells were cultured in complete IMDM at a concentration of 2 × 10^6^ cells/mL with the following conditions for 48 h in a 24-well plate: B cells cultured alone (naïve B), B cells stimulated with 2 μg/mL recombinant PD-L1-Fc (naïve B + PD-L1) (Biolegend, San Diego, CA, USA.), B cells stimulated with 1 μg/mL recombinant CD40L (PeproTech, Cranbury, NJ, USA) (activated B), and B cells stimulated with 2 μg/mL recombinant PD-L1-Fc and 1 μg/mL recombinant CD40L (activated B + PD-L1). Additionally, 50 ng/mL PMA, 500 ng/mL ionomycin, and 1 μM Monensin were added in each culture condition at the final 5 h of the culture.

### 4.6. Flow Cytometry

Cells were collected and washed with PBS (pH = 7.4). APC-labeled F4/80 (1:100, cat: 123116.) or CD19 (1:100, cat: 115512.), PE-labeled rat anti-mouse CD40L (1:100, cat: 157003.), Brilliant Violet 421-labeled rat anti-mouse PD-1 (1:100, cat: 109121.), or PD-L1 (1:100, cat: 124315) (all from Biolegend, San Diego, CA, USA) were used to label the membrane expression marker. For cytoplasm protein stain, B cells were fixed with 4% PFA at 4 °C for 15 min after the membrane antigens were labeled. The primary antibodies used for the cytoplasm protein were as follows: rat anti-mouse Ebi3 (1:150, cat: 210-501-B66, Rockland, ThermoFisher Scientific, Waltham, MA, USA), rabbit anti-mouse IL-12a (1:100, cat: BS-0767R, Bioss, ThermoFisher Scientific, Waltham, MA, USA), FITC conjunction rat anti-mouse TGF-β1 (1:100, cat: 141414, Biolegend, San Diego, CA, USA), and IL-10 (1:100, cat: 505006, Biolegend, San Diego, CA, USA). For non-fluorescence conjunction primary antibody, the Alex flour 488 conjunction goat anti-rabbit IgG antibody (1:2000, cat: ab150077, abcam, Cambridge, UK) and Alex flour 594 conjunction goat anti-rat IgG secondary antibody (1:2000, cat: ab150160, abcam, Cambridge, UK) were used to detect the primary antibody. A total of 10,000 cells were counted in each sample with BD LSR Frotessa X-20 (BD Biosciences, San Jose, CA, USA), and the data was analyzed using the Flowjo 10.9.0 software (Flow Jo, Ashland, OR, USA).

### 4.7. Immunofluorescence Staining

At the end of co-culture, B cells were collected and pelleted with a centrifuge. The B cells were fixed with 4% paraformaldehyde at 4 °C for 15 min, followed by washing with PBS for 5 min. Fixed B cells were diluted with 50 μL PBS after washing, and 10 μL of each sample was seeded on the slide and dried at 37 °C for 10 min. Additionally, 0.1% Triton-X100 was used for permeabilization at room temperature for 15 min. Cells were stained with rat anti-mouse EBi3 (1:150, cat: 210-501-B66, Rockland, ThermoFisher Scientific, Waltham, MA, USA) and rabbit anti-mouse IL-12a (1:100, cat: BS-0767R, Bioss, ThermoFisher Scientific, Waltham, MA, USA) overnight at 4 °C, and the Alexa flour 488-labeled goat anti-rabbit IgG (1:500) and Alexa flour 594-labeled goat anti-rat IgG (1:500) secondary antibodies were used to recognize the primary antibody. Pictures were taken with a Zeiss LSM 880 confocal system (Zeiss, Jena, Germany) at 63× with oil, and the average of positive cells from 10 views for each slide was counted per sample.

### 4.8. RT-qPCR

Total RNA was isolated with the PureLink^®^ RNA mini kit (Invitrogen, Carlsbad, CA, USA) following the manufacturer’s instructions. cDNA was synthesized with a Verso cDNA Synthesis Kit (ThermoFisher Scientific, Waltham, MA, USA). Subsequently, RT-qPCR was performed using PowerUp^TM^ SYBR^TM^ Green Master Mix (Applied Biosystems, Foster City, CA, USA) for qPCR on the QuantStudio 3 system (Applied biosystem, ThermoFisher Scientific, Waltham, MA, USA). Typical cycling parameters were used as follows: hold stage at 50 °C for 2 min, followed by 95 °C for 10 min; PCR stage (40 cycles) at 95 °C for 15 s, 60 °C for 1 min, and 95 °C 15 s; and melt curve stage at 95 °C for 15 s, 60 °C for 1 min, and 95 °C for 15 s. Each sample had three duplicates per gene. The 2^−ΔΔCT^ was used to calculate relative expression values. The primers used were as follows: *PD-1*: F: 5′-GCTCAACAAGTATGTCAGAGGC-3′; R: 5′-AGCTCCTCATAGGCCACACTA-3′, *GAPDH*: F: 5′-TGTGTCCGTCGTGGATCTGA-3′; R: 5′-TTGCTGTTGAAGTCGCAGGAG-3′.

### 4.9. Statistical Analysis

The data distribution was presented as mean ± SEM and analyzed using SPSS 23.0 and GraphPad Prism 8.0.2. The Shapiro–Wilk test and Levene’s test were used to assess the normal distribution and the homogeneity of variance of the data. Student’s *t*-test was used to analyze the difference between two independent groups, and a one-way analysis of variance followed by Tukey’s test or Dunnett’s *t*-test were used to carry out multiple data comparisons. Non-normal distribution data were analyzed with the Mann–Whitney U test. Statistical significance was set at a two-tailed *p* value < 0.05.

## 5. Conclusions

In summary, this study demonstrated for the first time that M2 macrophages promote IL-35^+^ but not TGF-β1^+^ regulatory B cell differentiation through the activation of the PD-L1 and PD-1 pathways in vitro.

## Data Availability

The data that support the findings of this study are available in the article. Further inquiries can be directed to the corresponding author.

## Supplementary Materials

The following supporting information can be downloaded at https://www.mdpi.com/article/10.3390/ijms26115332/s1.

## Abbreviations

The following abbreviations are used in this manuscript:

EAE	Experimental Autoimmune Encephalomyelitis
IMDM	Iscove’s Modified Dulbecco’s Medium
Breg	Regulatory B Cell
