# Supplementary material for: Pro-Resolving Macrophage-Induced IL-35+ but Not TGF-β1+ Regulatory B Cell Activation Requires the PD-L1/PD-1 Pathway
Source: Int J Mol Sci. 2025 Jun 1;26(11):5332. doi: 10.3390/ijms26115332 (PMC12155292; doi:10.3390/ijms26115332)
Supplement: Supplementary file 1 [file ijms-26-05332-s001.zip › ijms-3595017-supplementary/Supplementary Figure S1.pdf]

Supplementary Figure S1

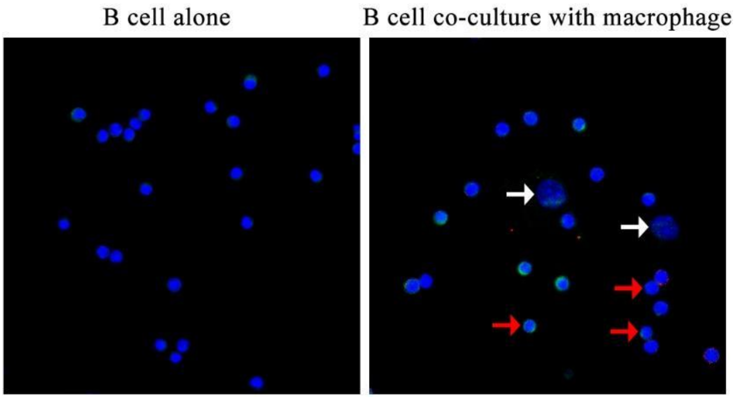

Supplementary Figure S1: B cells used for these co-culture experiments were isolated from splenocytes with ＞95% purity that were verified by flow cytometry using anti-CD19 antibody. Then the purified B cells were co-cultured with M0 macrophages for 48h. Then collected the unattached cells, followed by staining with IL-35(Ebi3^{+}IL-12a^{+}), green is IL-12a, red is Ebi3, red arrow is B cells, white arrow is Macrophage.
