# Supplementary material for: Pro-Resolving Macrophage-Induced IL-35+ but Not TGF-β1+ Regulatory B Cell Activation Requires the PD-L1/PD-1 Pathway
Source: Int J Mol Sci. 2025 Jun 1;26(11):5332. doi: 10.3390/ijms26115332 (PMC12155292; doi:10.3390/ijms26115332)
Supplement: Supplementary file 1 [file ijms-26-05332-s001.zip › ijms-3595017-supplementary/Supplementary Figure S2.pdf]

Supplementary Figure S2:

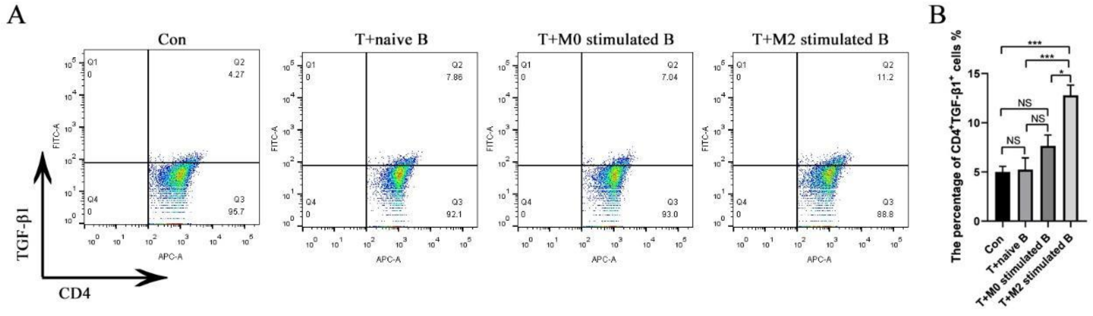

Supplementary Figure S2: M2 macrophage-induced Breg promote TGF-β1 expression in CD4$^+$ T cells. Spleen B cells and CD4$^+$ T cells were isolated with an immune microbead kit. B cells were cultured alone or co-cultured with M0/M2 macrophages for 48h. Then the B cells were co-cultured with CD4$^+$ T cells at the presence of 2μg/mL CD28 and 1μg/mL CD3 for 48h. The CD4$^+$ T cells were pre-treated with 2μg/mL CD28 and 1μg/mL CD3 for 24h. （A）The expression of CD4$^+$TGF-β1$^+$ T cells. (B) The statistics of CD4$^+$TGF-β1$^+$ (n=4). One-way analysis of variance followed by the Tukey test was used to assess multiple data comparisons. Data was shown as Mean±SEM. NS: no significant difference; * $P<0.05$； *** $P<0.001$.
