# Supplementary material for: Pro-Resolving Macrophage-Induced IL-35+ but Not TGF-β1+ Regulatory B Cell Activation Requires the PD-L1/PD-1 Pathway
Source: Int J Mol Sci. 2025 Jun 1;26(11):5332. doi: 10.3390/ijms26115332 (PMC12155292; doi:10.3390/ijms26115332)
Supplement: Supplementary file 1 [file ijms-26-05332-s001.zip › ijms-3595017-supplementary/Supplementary Figure S3.pdf]

Supplementary Figure S3

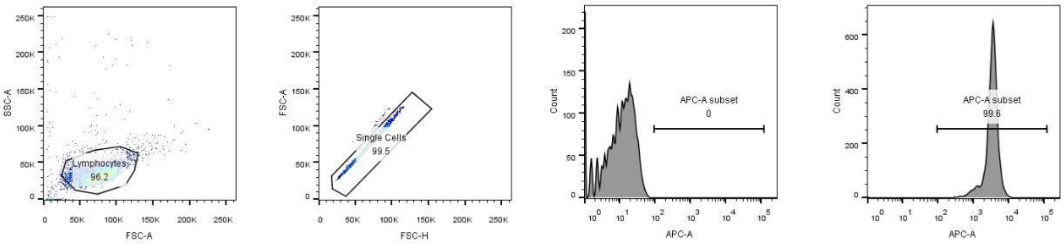

Gate on whole cell after isolation | Single cell | No stain control | APC-CD19 positive cell

Supplementary Figure S3: Spleen B cells were isolated with a Pan B cell isolation kit, and the purity of the CD19$^+$ B cells was detected by flow cytometry which was stained with an APC-CD19 antibody. The percentage of CD19-positive B cells was 99.6%.
